# Mr. Williams, on the Functions of the Liver

**Published:** 1807-06

**Authors:** J. Williams

**Affiliations:** Redruth


### *To the Editors of the Medical and Physical Journal.*

GENTLEMEN,

MANY have been the conjectures by able anatomists, on the use of the Liver, and, though several eminent men have exercised their extensive abilities, with a view to explain the utility of this important viscus, it still lies unexplored, and there remains a total ignorance among Physicians, as to the primary cause of its operation.

That the liver is the place wherein the bile is secreted, appears without a doubt, and it is almost universally allowed among medical men, that its express use is to assist with the pancreatic juice, in promoting the separation of the chyle from the feculent excrements; but when we consider the large quantity of bile which enters the duodenum, and its arrival in this gut after the pancreatic juice has almost extracted the chyle from the chyme, and from thence is by some supposed to separate the less nutritious parts from the most feculent, we are led to suppose it for a different purpose; for can we rationally imagine the Almighty Being has set aside such a large proportion of the human frame as the liver, and the extraction of so much bile from the blood, merely for the sake of assisting in chylification? or by some, the insignificant office of separating the few particles of chyle which were thought unworthy to accompany the pancreatic juice in its retreat to the lacteals? Had this been the case; I say, had there been no other use for the bile than assisting in chylification, man would be better without the addition of the liver to the human frame; for allowing the bile to separate half the chyle, which by all accounts it does not, the exhaustion of the system by the excretion of the bile would then be more than adequate to the nutritious power of the chyle secreted; so that he who supposes the excretion of the bile for no other purpose than assisting in chylification, may be compared to a person who endeavours to fill a reservoir by extracting from it a greater stream than it receives.

Dr. Rush, in your Journal for September, has given us an interesting hypothesis on the use of the liver, which he supposes is to anamalize the imperfect chyle that had not been formed into red blood, in its circulation through the lungs, or whatever part chyle is formed into blood, so as to to throw it into the duodenum to be taken up a second time through the lacteals, and by that means to save a great part of our nourishment, which would otherwise be lost to the system. That the liver prepares the imperfect chyle I allow, but for what purpose I shall endeavour to show in the following lines.

CHYLE, when introduced into the sanguiferous system, undergoes a particular process before it is converted into blood. It is generally the case, that any one substance formed from another, does not take to itself the whole of that substance from which it is formed; *id est,* one substance is not entirely changed into another, but it leaves behind some heterogeneous matter which is unfit for the ends it was designed. For instance, our food, when taken into the stomach, is not entirely fit for nourishment, but by undergoing different processes the feculent is separated from the nutritious; the former to be expelled from the system through the intestines, while the latter is conveyed to its future destination. Now, if such be the case with the chyle, which in my belief it is, there would be an indispensable necessity for some viscus, by the means of which that heterogeneous matter left in the system in the operation of sanguification should be expelled. For this do I conceive to be the use of the liver; not that I mean the operation of the liver is entirely confined to the carrying off the matter that had been introduced into the system with the chyle, and which is unfit for sanguification, but also for the excretion of any impurities mixed with the blood. For when we reflect on the absorption of matter which must naturally take place in the small-pox, cutaneous diseases, and backened swellings of all kinds, it would be irrational to suppose that this matter can continue in the system without producing several disagreeable diseases, and therefore we see the necessity of some particular viscus, by which our blood can be cleansed from all impurities, and thereby to keep our bodies in a better state of health than would otherwise be the case.

Another important use I ascribe to the liver is, when the blood vessels are over supplied with chyle from the lacteals, on account of a person taking more food than necessary; I say, its use is of great importance here, as it opens a friendly door to receive the superabundant chyle, so as to deposit it in the intestines, either to be taken up again when wanted, or to be immediately expelled per anum; the latter of which, I think, more likely, as we are generally liable to diarrhœas after having lived for some time more freely than accustomed. Therefore, we

 perceive perceive the indispensable necessity of this valuable viscus; without which, our blood vessels would not only be over distended or ruptured, but suffocation itself would be the immediate consequence of our taking so much more food than necessary.

By this means also we can perceive, why people accustomed to eat a great quantity of food are not at all times liable to obesity, but rather to marasmus, as the liver, by the extension of its excretory ducts, being prompt to excrete more nourishment from the blood than is necessary for the production of health. So also from the above, we not only conceive the liver to be the viscus wherein the blood is cleansed from its impurities, but that it is also the means by which the lives of many persons, particularly children, have been preserved by the expulsion of the superabundant chyle from the system. Hence also may be the reason why children are endowed with larger livers than men, in proportion to their size, as they are generally subject to more intemperies; also by their food being in a more fluid state than ours, more heterogenous particles may be conveyed into their system. Likewise in long continued fevers, where the patient has not received any nourishment for several days, we sometimes find copious discharges per anum. Now, as it is impossible that it could have come from the stomach, it certainly must have taken its descent from the liver. For what purpose? Not merely for the sake of assisting in chylification, as there is no chyme in the intestines on which it may operate, but the blood being driven through the system with greater velocity than usual, carries along with it every particle it meets in its way, which is excreted by the liver, and forms this discharge; hence may be the cause why a person is frequently hearty and subject to high health after a fever.

I might produce the history of several diseases attendant on the obstruction of the liver, in order to substantiate my opinion; such as phthysis, phrenitis, dyspepsia, suffocation, &c. all of which are frequently produced by drinking strong spirituous liquors, so as to injure the said viscus. The three former of which, namely phthisis, phrenitis, and dyspepsia, are occasioned by the blood being thrown into the lungs or brain in its impurified state, carrying along with it some acrid substance, taken up in its passage through the different parts of the body, and which, owing to the diseased state of the liver had not been excreted, it stagnates there, and produces inflammation, tion, &c. The latter, suffocation, may be occasioned by a person drinking to excess strong spirits after making a very hearty meal, so as to obstruct the liver when most wanted; that is, the system being over distended with chyle, the effects of so large a quantity of food digested, endeavours to throw off its load by the excretion of the liver; but on its arrival to that viscus, it finds it weakened by the spirits, and thus frequently occasions suffocation, coma, &c. It is a common case to find men dead the following morning, after having eaten and drank to excess the preceding day. But as it is not my intention, neither is it consistent with the rules of your Journal, to trouble you with all the corroborants of this subject, I shall therefore conclude, by making a few observations on the operation of mercury on this valuable viscus.

It is a well known case, that mercury has a more particular influence over the liver, than any other part of the human frame; but how to account for this, appears to be disputed among medical men.* Some have recommended it to be rubbed on the epigastric region, merely for the sake of friction on the parts affected.† Another has thought adviseable to rub it on the same region, because he supposes it nearly connected with the liver by means of its absorbents, and by that means is sooner conveyed to the said viscus; but we generally perceive that whether it be produced in the system by the means of friction, or whether it be taken in the stomach, its effect on this viscus is nearly the same. Now, if mercury introduced into any part of the sanguiferous system, has a particular tendency to affect the liver, it must be because the blood undergoes a more particular investigation in this viscus than it does in any other: the explanation of which will corroborate my opinion as to the *use of the Liver*: the mercury being driven through the system, like any other foreign substance is excreted; in the operation of which, the liver, on account of the deobstruent quality of the mercury, is cleansed from all its obstructions.

I have seen a phrensy removed by the administration of five grains of calomel; but unfortunately, the same means which occasioned an inflammation of the brain, also produced an inflammation of the lungs, which ended in sup-

 puration,

* Drs. Saunders and Pemberton.

† Dr. Stone.

puration, and the same person is now in the last stage of consumption.

From the above, we can in some measure perceive why mercury has often been efficacious in cutaneous diseases, and also why it has in some degree proved an efficacious medicine in consumption.

I am, &c.

*Redruth, April 7,* 1807. J. WILLIAMS.

